# New Editor-in-Chief, New Challenges

**DOI:** 10.5935/abc.20180012

**Published:** 2018-01

**Authors:** Carlos Eduardo Rochitte

**Affiliations:** Instituto do Coração - InCor; Hospital do Coração - HCOR, São Paulo, SP - Brazil

**Keywords:** Periodicals as topic/history, Periodicals as Topic/trends, Journal Impact Factor, Publishing/trends

It was a great honor and privilege to be appointed to serve as the new editor-in-chief of the *Arquivos Brasileiros de Cardiologia* for the 2018-2021 period. In this first editorial, I would like to thank my peers and colleagues, who have manifested sincere and total support to my indication. It is undoubtedly a great challenge to contribute to the most important scientific journal of Cardiology in South America.

Moreover, it is on the shoulders of the previous editors-in-chief that I humbly make myself available to collaborate with this communication channel of the Brazilian Cardiology. The work here developed is monumental and was only possible because of the collaboration of the associate editors and reviewers, who are part of the great family of the *Arquivos Brasileiros de Cardiologia*. And it will go on like this. Thus, I ask for the support of all involved in the task in the coming years.

**Figure 1:**
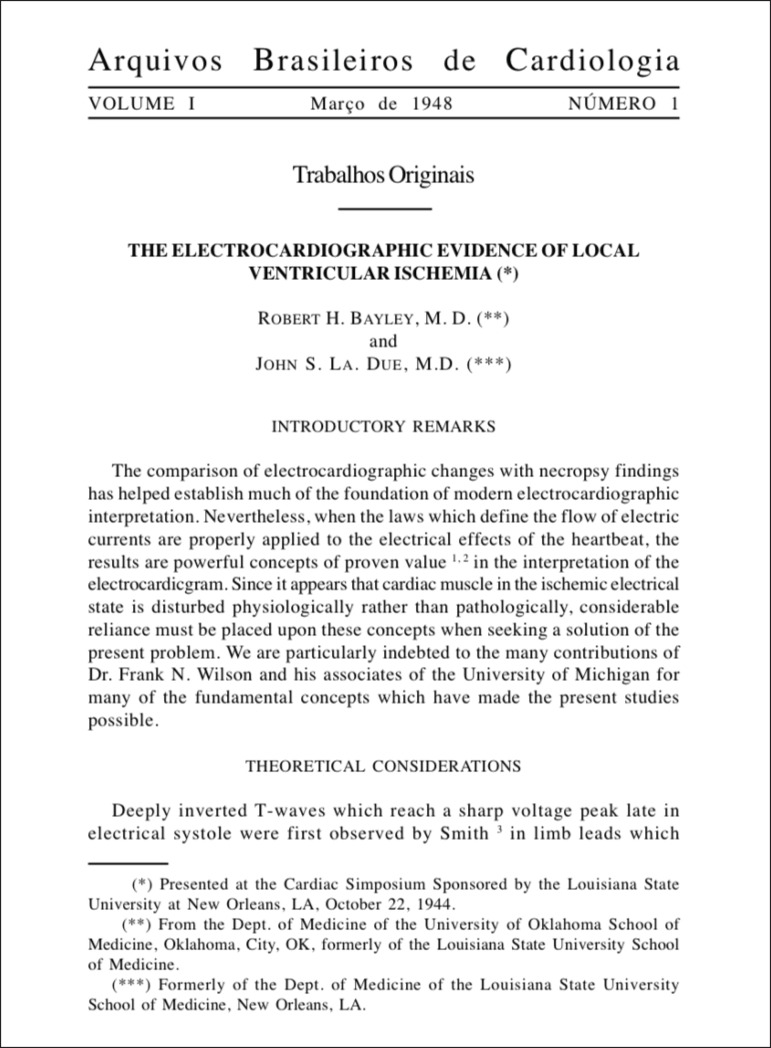
First page of the first article published by the Arquivos Brasileiros de Cardiologia in 1948.

The constitution of a writing committee for the Brazilian Society of Cardiology journal was proposed by Dante Pazzanese and Luiz V. Décourt, and the first “director” of the *Arquivos Brasileiros de Cardiologia* was Dr. Jairo Ramos, who suggested the journal’s name, which has persisted since 1948. The histories of the Brazilian Cardiology, of the Brazilian Society of Cardiology and of the *Arquivos Brasileiros de Cardiologia* have mingled for decades. It is worth noting that the first study published, entirely written in English, was "*The electrocardiographic evidence of local ventricular ischemia*", by Robert H. Bayley and Jolm S. La Due, from Oklahoma City, United States (Figure 1).^1^ This clearly shows that the internationalization potential of the *Arquivos Brasileiros de Cardiologia* has always been in its DNA. It is up to us, as a Society of Cardiology, to fully develop it.

I am especially grateful to our former editor-in-chief Dr. Luiz Felipe P. Moreira for its excellent management and for handing on a well-structured and organized journal to me. His editorial from last December revisits the accomplishments of the past eight years, a period when I was associate editor of diagnostic and imaging methods.^2^ What I learned during his management, as well as his support, substantiated my decision of applying for editor-in-chief of the *Arquivos Brasileiros de Cardiologia*.

Several actions have been planned to speed up the article review process of our journal and make it more attractive to authors. With the constant progress of science and of its outreach channels, the *Arquivos Brasileiros de Cardiologia* need to be prepared to keep up with such changes and innovations.

In the 2018-2021 period, two master beams will provide the base for that innovation: our journal’s internationalization and its impact factor improvement. Those two master beams were chosen because of the stability, in the past years, of our journal’s impact factor slightly above 1 and non-adherence to the internationalization issues recommended by Scielo.

The first master beam has been championed by Scielo for a while and will be emphasized in coming the championed years. One aspect of internationalization is the participation of international associate editors. In 2018 we will begin with two new international editors and one international co-editor, thus meeting the Scielo recommendation of having approximately 30% of international associate editors. We aim at getting more international visibility and attracting “good science” for the *Arquivos Brasileiros de Cardiologia* in the form of original articles. Review articles will maintain the tradition of reviewing Cardiology topics and its limits with other specialties, always indicating the future steps in the area, such as the review article of the January issue.^3^ We are renewing our associate editors, the international reviewers and our editorial board aiming at both speeding up the publication of articles in the *Arquivos Brasileiros de Cardiologia* and improving their quality.

International collaboration has been a mechanism to enhance the impact factor of some European international journals. I believe we need to refine our performance in that area, having, thus, to adopt internationalization measures for the *Arquivos Brasileiros de Cardiologia*. Our two major objectives are obviously synergistic.

In addition, measures to speed up and modernize the article review process are programmed, in an attempt to rapidly notify the authors about the acceptance or refusal of their articles in the *Arquivos Brasileiros de Cardiologia*. We want to increase the satisfaction of the authors and reviewers during the editorial processes in our journal. The first important step is the adoption of a new electronic system of submission, most likely the *ScholarOne*, which will allow a faster and more practical management of the articles from the viewpoints of all involved: authors, associate editors, editor-in-chief, reviewers and editorial assistants. In addition, we will work close to authors and reviewers to obtain a rapid and effective review, allowing the editors to decide more accurately and efficiently.

Some changes in the formats of the articles are being planned to make them more concise, direct and pragmatic. In a recent visit of Prof. Valentin Fuster, JACC’s editor-in-chief, we asked “*How do you do it?*”, and the answer was “*Keep it simple!!*”. I believe we should listen to that advice and follow it. Thus, mini-editorials for the original articles and a Figure 1 summarizing the article are formal changes to be implemented.

However, not everything is planned linearly as described above. New challenges will ask for the participation of all players in this scientific process, including our Brazilian Society of Cardiology members. In a recent meeting, Scielo has determined new guidelines to be followed by Latin American journals, some of which with disruptive characteristics and unpredictable final effects. The most important examples are the continuing publication and the concept of “open science”. The latter includes the publication in repositories of source data that generated the manuscript’s results. Such publication makes data public and available to be used, checked and re-analyzed by groups other than the original authors. Although extremely controversial, that mechanism seems to increase the number of citations of the articles, and, thus, the impact of the articles and of the journal, in addition to adding credibility to them. Would this function in the same way in the Brazilian scientific environment? This response can only be provided by the scientific community, and its adoption by the *Arquivos Brasileiros de Cardiologia* has to undergo a deep and thorough discussion. Likewise, and possibly even more controversial, “open science” proposes accepting the articles in the “preprint” format. Briefly, there are online repositories that accept scientific articles before undergoing peer review. This ensures the authors maintain “property” of the idea and data immediately, allowing them to be cited by other authors, but this can generate the exposure of low-quality articles. However, during exposure, similarly to an Internet forum, comments can be made, and the authors can use them to improve their publication quality. Several journals already accept the submission of articles that had been published as preprint. Should the *Arquivos Brasileiros de Cardiologia* accept that too? Again, this has to be thoroughly discussed, and we have to face a new challenge to adapt to the new digital reality of our virtual world. Scielo seems to strongly support those measures that will soon be mandatory. There is evidence in the literature that the movement towards “open science” increases the impact factor of the journals.^4,5^ I believe we have to go along with the change of times.

In addition, we have planned to use more intensively the social media to disseminate the *Arquivos Brasileiros de Cardiologia* content. There is evidence in the literature that the presence of journals in Twitter significantly increases the number of citations of articles and their impact.^6^

However, some things never change, and the “good science” and the relevance of the articles continue to depend on traditional scientific aspects, such as the changes in clinical practice and the generation of new knowledge or ideas on the pathophysiology, natural history or treatment of a disease. Based on the “good science” that has been fostered by the *Arquivos Brasileiros de Cardiologia* over seven decades of existence, and because it represents the science of one of the major societies of Cardiology in the world, I am sure that the future of our *Arquivos Brasileiros de Cardiologia* is brilliant and will continue to merge with the history of the Brazilian Cardiology.
